# Stabilization of an Enantiopure Sub‐monolayer of Helicene Radical Cations on a Au(111) Surface through Noncovalent Interactions

**DOI:** 10.1002/anie.202103710

**Published:** 2021-06-08

**Authors:** Niccolò Giaconi, Andrea Luigi Sorrentino, Lorenzo Poggini, Michela Lupi, Vincent Polewczyk, Giovanni Vinai, Piero Torelli, Agnese Magnani, Roberta Sessoli, Stefano Menichetti, Lorenzo Sorace, Caterina Viglianisi, Matteo Mannini

**Affiliations:** ^1^ Department of Chemistry “Ugo Schiff” and INSTM Research Unit University of Florence Via della Lastruccia 3–13 50019 Sesto Fiorentino Italy; ^2^ Department of Industrial Engineering and INSTM Research Unit University of Florence Via Santa Marta 3 50139 Florence Italy; ^3^ Istituto di Chimica dei Composti Organometallici (ICCOM) CNR Via Madonna del Piano, 10 50019 Sesto Fiorentino Italy; ^4^ Istituto Officina dei Materiali (IOM) CNR, Laboratorio TASC Area Science Park, S.S. 14 km 163.5 34149 Trieste Italy; ^5^ Department of Biotechnology, Chemistry and Pharmacy and INSTM Research Unit University of Siena Via Aldo Moro 2 53100 Siena Italy

**Keywords:** chirality, helicenes, organic radicals, spinterfaces, X-ray natural circular dichroism

## Abstract

In the past few years, the chirality and magnetism of molecules have received notable interest for the development of novel molecular devices. Chiral helicenes combine both these properties, and thus their nanostructuration is the first step toward developing new multifunctional devices. Here, we present a novel strategy to deposit a sub‐monolayer of enantiopure thia[4]helicene radical cations on a pre‐functionalized Au(111) substrate. This approach results in both the paramagnetic character and the chemical structure of these molecules being maintained at the nanoscale, as demonstrated by in‐house characterizations. Furthermore, synchrotron‐based X‐ray natural circular dichroism confirmed that the handedness of the thia[4]helicene is preserved on the surface.

## Graphical abstract

A strategy based on noncovalent interactions has been developed to deposit a sub‐monolayer of enantiopure thia[4]helicene radical cations on a pre‐functionalized Au(111) substrate. This approach results in both the paramagnetic character and the chirality of the molecules being retained at the nanoscale.

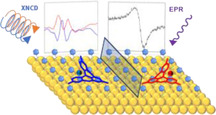

Open‐shell organic molecules constitute a remarkable platform both for the study of their fundamental properties and for applied research. Their nanostructuration is of the utmost importance for controlling and exploiting their properties at the nanoscale, either as individual objects or as an ensemble regularly arranged in 2D structures. In these nanostructures, the paramagnetic centers can be addressed individually using local probes or collectively in an ensemble molecular junction.^[1]^ The latter approach is already in use for the development of several classes of (opto‐)electronic devices, such as organic light‐emitting devices, dye‐sensitized solar cells, and organic spin valves.[^2^, ^3^, ^4^] The use of organic radicals may influence the performances of these devices, some of which are already in use in our daily lives.^[5]^ Actually, organic radicals have been proposed as building blocks for several multifunctional devices[^6^, ^7^] and, in particular, as spin filters in molecular spintronic devices[^8^, ^9^] because of their relatively long spin coherence length. The nanostructuration of several families of organic radicals has been investigated for this purpose, including perchlorotriphenylmethyl, 1,3‐bis(diphenylene)‐2‐phenylallyl, 2,2,6,6‐tetramethyl piperidine 1‐oxyl, and nitronyl‐nitroxide radicals.[^10^, ^11^, ^12^, ^13^, ^14^] Extra advantages^[15]^ can be foreseen by the use of helicene radicals, in which the paramagnetic character is associated with the structural chirality^[16]^ of the individual molecules. This opens the possibility of tailoring the spin filtering by exploiting the chiral‐induced spin selectivity (CISS) effect.[^17^, ^18^, ^19^]

Herein, we report the first successful attempt of depositing a chiral organic radical: a thia[4]helicene radical cation^[20]^ was assembled on a thiophenol‐coated Au(111) substrate through a multistep wet chemistry approach. We selected this compound because it combines a stable chiral structure^[20]^ with paramagnetic properties, thus being a promising candidate for developing multifunctional spintronic devices. A detailed spectroscopic characterization confirmed the retention of the chemical structure and the radical nature after the deposition process. Furthermore, by performing X‐ray natural circular dichroism (XNCD) measurements, we probed the persistence of the handedness of molecules assembled on the surface, thereby confirming that an enantiopure compound can be assembled on the substrate and the racemization process avoided.

The thia[4]helicene radical cation (3,7,11‐trimethyl[1,4]benzothiazino[2,3,4‐*kl*]phenothiazine hexafluoroantimonate radical cation, **RadE**; Figure 1) was synthesized by following the previously reported procedure.^[20]^ Substrates were obtained by thermal evaporation of 120 nm gold on muscovite mica followed by a hydrogen flame annealing treatment to induce the reconstruction of the Au(111) surface.^[21]^ **RadE** was assembled on the surface by performing the two‐step wet chemistry approach depicted in Figure 1. The first step involved the direct incubation of the cleaned substrate in a highly diluted solution of thiophenol in EtOH to induce the formation of a self‐assembled monolayer (SAM) on the gold. Then, after rinsing the surface with pure solvent, the thiol monolayer was incubated in a dilute solution of **RadE** in CH_2_Cl_2_.

**Figure 1:**
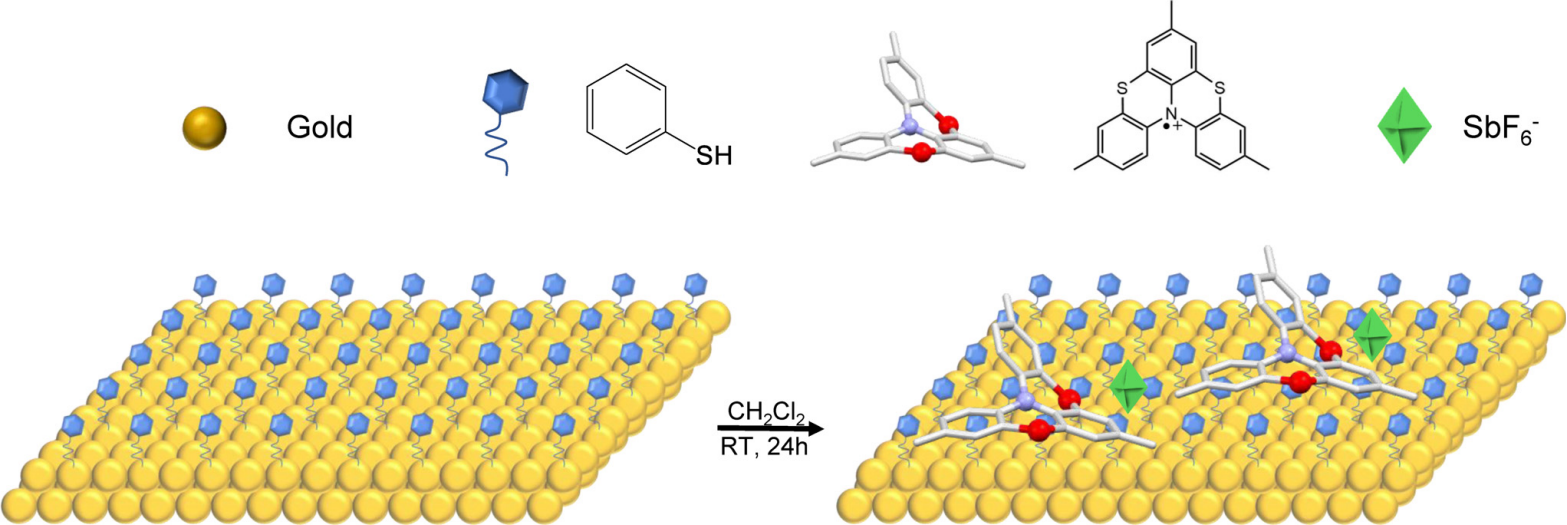
The deposition of **RadE** on thiophenol‐templated Au(111). The gold substrate was incubated for 24 h in thiophenol and then the resulting thiophenol template was incubated for an additional 24 h in the solution containing the radical cation. The monolayer was assembled by exploiting the electrostatic interaction between the positive charge of the helicenes and the electron density of the aromatic rings of the template. Color code: sulfur, red; nitrogen, violet; gold, yellow; carbon, light gray; hexafluoroantimonate, green.

Several cleaning cycles using pure solvent were performed at the end of the entire deposition process to guarantee the removal of excess molecules and leave only a sub‐monolayer of **RadE** directly interacting with the thiophenol layer. Pre‐functionalization with thiophenol was adopted to avoid the direct interaction of the radical backbone with the gold surface, which was expected to be detrimental.^[22]^

The following in‐house characterizations were performed on samples assembled starting from a racemic mixture of **RadE**. The radical cations monolayer assembled on thiophenol were characterized by X‐ray photoelectron spectroscopy (XPS).

S 2p, F 1s, and N 1s (Figure S1) signals were considered in a semiquantitative analysis to estimate the presence of these elements in the deposited molecules (radical cations and counterions) on the substrate. A crucial piece of information regarding the presence of the molecules on the surface can be deduced by analysis of the S 2p region in the XPS spectra of the bulk sample (**RadE**), of a thiophenol monolayer (**TP@Au**), and of helicene radical cations assembled on a thiophenol monolayer (**RadE_TP@Au**; Figure 2).

**Figure 2:**
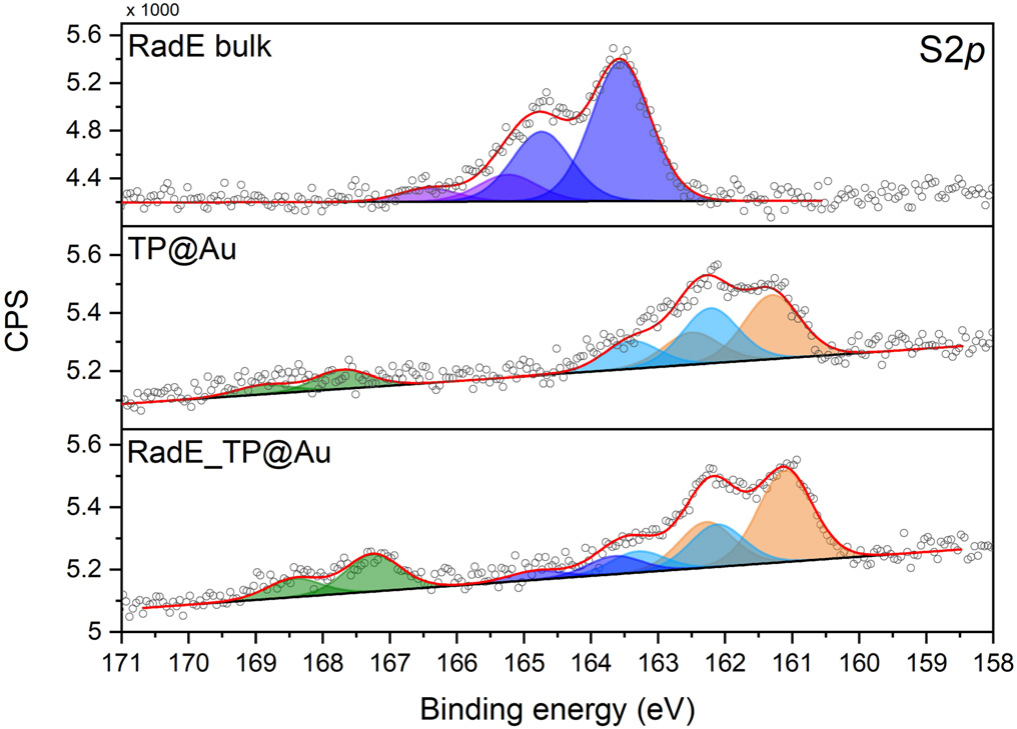
S 2p regions of bulk **RadE**, **TP@Au**, and **RadE_TP@Au**. Assignment of the best‐fitting components: S atoms of the helicene backbone: blue; S‐bound Au substrate: orange; S atoms of thiophenol molecules physisorbed on the surface after the rinsing procedure: cyan; S atoms oxidized after exposure to the air: green. Each signal is accompanied by its spin‐orbit‐coupled S 2p_1/2_ component shifted by 1.2 eV.

The **RadE** bulk sample features a major component at 163.5 eV, which is attributable to sulfur atoms of the helicene structure. In the case of **TP@Au**, the signal at 161.3 eV confirms the formation of bonds between the gold and the sulfur atoms of the thiophenol. We also detected the presence of another component at 162.2 eV, which arises from the physisorbed molecules left on the surface even after the rinsing procedure (ca. 32 %). A signal at a higher binding energy (ca. 168 eV) shows that a minor fraction (ca. 15 %) of molecules is oxidized, either during the deposition process or on exposure during sample manipulation (see Table S1). The component at 163.5 eV present in the spectra of **RadE** cannot be detected here, since the helicene sulfur atom is not present in **TP@Au**. The S 2p region of the **RadE_TP@Au** sample features all the components observed in the spectra of the two above‐described systems. Indeed, in addition to the signals of the thiophenol, we observe the presence of components attributable to **RadE** at 163.5 eV. Considering the experimental error of the XPS technique, we can confirm that the procedure adopted for the deposition avoids any changes in the molecular structure (see Table 1).

**Table 1:** Elemental analysis of the thia[4]helicene radical cation assembled on thiophenol‐templated Au(111).

Sample	S 2p [%]	N 1s [%]	F 1s [%]
expected stoichiometry	22.2	11.1	66.7
bulk	25.4±1.3	11.6±0.6	63.0±3.2
**RadE_TP@Au**	24.2±1.2^[a]^	13.5±0.7	62.2±3.1

[a] Only the contribution from **RadE** at 163.5 eV (blue component in Figure 2) has been included to properly evaluate the stoichiometry of the physisorbed material on top of the thiophenol monolayer.

The number of molecules of **RadE** adsorbed on the thiophenol‐functionalized surface can be estimated by using the integrated intensity of the S‐Au component in the XPS spectra of the aromatic thiol as a reference for one molecular layer. Literature reports^[23]^ indicate a packing density of 4.3 molecule/ nm^2^ for a monolayer of thiophenol on Au(111). Considering that each **RadE** molecule contains two sulfur atoms and the integral ratio of the two signals is about 5:1, we can estimate a **RadE** coverage of 0.43 molecule/nm^2^. According to crystallographic data,^[20]^ each RadE molecule occupies a surface area of about 1.0 nm^2^, thus confirming that the adopted procedure led to a sub‐monolayer deposition of the radical on the thiophenol monolayer. It is worth mentioning that, from a qualitative point of view, the N 1s region does not allow any relevant information to be obtained because of the sensitivity of the radical moiety to X‐rays in the molecular monolayer. The presence of nitrogen atoms in different oxidation states after exposure to X‐rays and secondary electrons (see Figure S1) is indeed confirmed by the several components needed to properly fit the experimental data. This is in agreement with previous reports indicating that the radical function of monolayer deposits cannot survive treatment with X‐rays.[^14^, ^24^, ^25^] It is important to stress here that, due to this alteration, the previous characterization, as well as the following one based on synchrotron light, provide only information about the molecular backbone structure. Alternative techniques have been used to confirm the survival of the radical function in the monolayer.

Time‐of‐flight secondary ion mass spectrometry (ToF‐SIMS) corroborates the stability of these molecules on the surface. This characterization was carried out on bulk **RadE**, **TP@Au**, and **RadE_TP@Au**. The most relevant signals were detected in the positive ion spectra: Figure 3 a shows the region between *m*/*z*=300 and 360, where one expects the most significant signals for the molecule fragmentation (see Table S2 for the assignment of the positive ion signals for the three samples). A high‐intensity signal at *m*/*z*=347 that is attributable to the radical cation is detectable in the **RadE** sample. Its isotopic distribution pattern is in perfect agreement with the theoretically expected distribution. Spectra obtained on **TP@Au** feature a signal occurring at *m*/*z*=306, consistent with the fragment [C_6_H_5_SAu]^+^, thus confirming the formation of the S−Au bond. A larger number of signals were observed for the **RadE_TP@Au** monolayer deposits as a result of a more efficient fragmentation of the sample with respect to the bulk sample. In this case, the spectrum is dominated by signals at higher *m*/*z* values, which can be attributed to fluorine‐containing fragments related to the counterion. It is worth noting that, despite being less intense, the two main signals observed in **RadE** and **TP@Au** are still detectable, thus confirming that the molecule and the initial passivating agent of the gold surface are intact.

**Figure 3:**
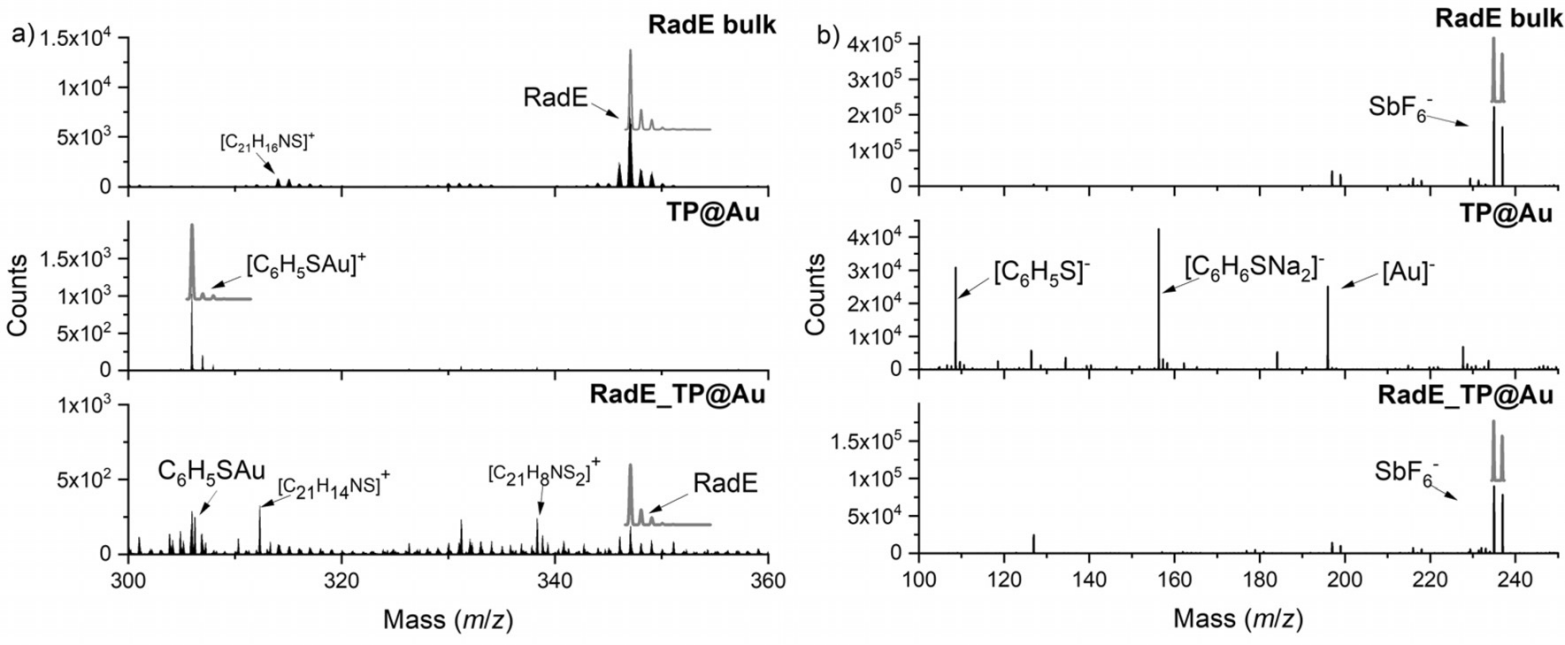
a) ToF‐SIMS positive‐ion spectra of bulk **RadE**, **TP@Au**, and **RadE_TP@Au** in the region *m*/z=300 to 360. b) ToF‐SIMS negative‐ion spectra of bulk **RadE**, **TP@Au**, and **RadE_TP@Au** in the region *m*/*z*=100 to 250. Gray lines above the experimental data represent the theoretical isotopic distribution of the most significant signals.

Some interesting information can also be obtained from the negative ion spectra (Figure 3 b). As expected, the bulk sample features only one prominent signal at *m*/*z*=234, which is assigned to the SbF_6_^−^ counterion. For the **TP@Au** sample, in agreement with previous reports on a thiophenol self‐assembled monolayer,^[26]^ signals arising from clusters with the general formula [(C_6_H_5_S)_*n*_Au_*n*−1_]^−^ (*n*=1–4) are observed. The **RadE_TP@Au** spectra show only one significant signal at *m*/*z*=234. The presence of both SbF_6_^−^ and **RadE** signals in the negative and positive ion spectra of the **RadE_TP@Au** samples gives evidence that we have deposited intact molecules on top of the thiophenol SAM (see Figure S2 for ToF‐SIMS spectra over a wider range).

X‐band (frequency ca. 9.4 GHz) EPR spectroscopy has been used to verify whether the deposition process influences the retention of the radical properties of the helicenes once assembled on the surface and to obtain information about the interaction among the paramagnetic centers within the monolayer.

The spectrum acquired for **RadE_TP@Au** (Figure 4) at 30 K features a clear signal with a line shape typical of a quasi‐isotropic *S*=1/2 paramagnetic system, closely resembling the EPR spectrum of the powder sample (Figure 4). Despite the low amount of material (ca. 10^13^ spin), which is close to the sensitivity limit of the technique, we have been able to collect meaningful spectra. For comparison, literature reports on EPR spectra of fluid solutions of **RadE** ^[20]^ show a complex hyperfine structure, while the spectrum of a frozen solution shows well‐resolved *g*‐anisotropy (see Figure S3). This indicates that when molecules are assembled in the monolayer, the 2D intermolecular exchange interaction is strong enough to wash out the hyperfine structure and average out the anisotropic features.^[27]^ The similarity with the spectrum of the powder sample further suggests a comparable magnitude of intermolecular exchange interaction in the solid state and in the monolayer. At the same time, angular‐dependent measurements of the SAM spectra did not provide evidence of any clear variation with field orientation (Figure S4). Since single‐crystal spectra of the pure sample clearly show the persistence of angular dependence (Figure S4), we can rule out long‐range structural order in the SAM.

**Figure 4:**
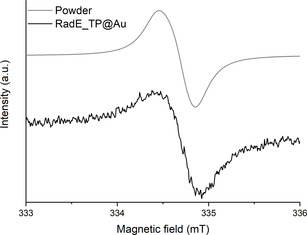
X‐band EPR spectra of **RadE** powder (gray) and **RadE_TP@Au** (black) at 30 K.

Once the feasibility of the surface assembly of the racemic mixture of **RadE** had been demonstrated, the same procedure was repeated for the two enantiopure compounds **(P)‐RadE** and **(M)‐RadE** (see inset of Figure 5), which were separated using HPLC.^[28]^ Samples of SAMs of the two enantiomers were characterized by X‐ray natural circular dichroism (XNCD).[^29^, ^30^] The selectivity of the core electron excitations allows investigation of the contribution of each element to the chirality of the molecule. Combining this feature with the surface selectivity makes XNCD an excellent technique to investigate chirality at the nanoscale.^[31]^ Absorption spectra were acquired by thermalizing the sample at 100 K to reduce radiation damage and with an angle of 45° between the beam and the normal to the surface of the sample. We focused on the carbon K‐edge since the chiral character of **RadE** lies mainly in the carbon atoms that constitute the helical structure of the molecule, which are expected to give different absorption contributions under circularly polarized light.

**Figure 5:**
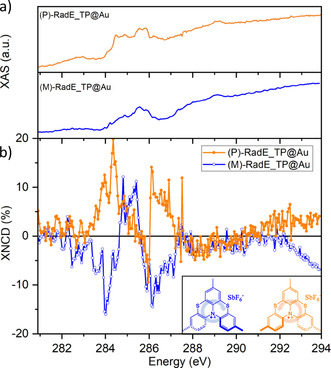
a) Isotropic XAS spectra at 100 K at the carbon K‐edge energy of **(P)‐RadE_TP@Au** and **(M)‐RadE_TP@Au**. b) XNCD spectra of the two enantiopure samples. Inset: molecular structures of **(P)‐RadE** and **(M)‐RadE**.

The isotropic XAS spectra (Figure 5 a), obtained by averaging the circular absorption cross‐sections for left (σ^L^) and right (σ^R^) circularly polarized light of both enantiomers, show spectral features attributable to both the C 1s→σ* transition at about 289.4 eV and to the C 1s→π* transition at a lower binding energy. The XNCD signals, evaluated as (σ^L^–σ^R^) and normalized to the carbon K‐edge signal, acquired on a reference substrate (see Experimental Section in the Supporting Information for further details) are reported in Figure 5 b. The XNCD spectra of **(P)‐RadE_TP@Au** and **(M)‐RadE_TP@Au** clearly show signals of the same magnitude and opposite sign for the two enantiomers. This confirms the persistence of handedness of the molecules on the surface, in agreement with the relatively high racemization barrier of these molecules.[^28^, ^32^] Furthermore, the most intense signals detected at about 284 eV and 286.2 eV show strong dichroism, with a surprisingly high magnitude of about 10 % accordingly to the normalization approach adopted here (see the Supporting Information). Such high dichroism might be due to the intrinsic chiral character spread over the entire structure of the helicene radical cations,^[31]^ in contrast to systems where the chirality is attributable to the presence of a single stereogenic center.^[33]^ This strong effect might also originate from a spin filtering of electrons caused by selective electron emission (that the TEY detector is indirectly monitoring as an electrical defect) induced by chiral structures, following the selective absorption of light with a specific helicity. This process could occur through a spin‐selective intramolecular transport process, in a similar way to what was reported for a chiral polypeptide monolayer embedded in a hybrid system containing quantum dots and a Ni surface.^[34]^ A complete understanding of this phenomenon will require further studies, but the major result is the unambiguous detection of chirality on a sub‐monolayer deposit.

In summary, we have developed a deposition process for sub‐monolayers of chiral radical cations. We demonstrated that the assembly of these molecules by a wet chemistry approach does not cause any changes in the chemical structure or magnetic properties with respect to the bulk phase. The exploitation of complementary spectroscopic techniques such as XPS, EPR, ToF‐SIMS, and XAS allowed an unprecedented in‐depth investigation of the properties of this system, with a particular focus on the detection of natural dichroism at the carbon K‐edge induced by chiral structures in a molecular monolayer. The success of the assembly on the surface represents a fundamental step in the rational design of new spinterfaces, the active interlayer in molecular spintronic devices. For such an application, the use of chiral paramagnetic molecules will lead to extra control in spin injection processes.

## Conflict of interest

The authors declare no conflict of interest.

## Supporting information

As a service to our authors and readers, this journal provides supporting information supplied by the authors. Such materials are peer reviewed and may be re‐organized for online delivery, but are not copy‐edited or typeset. Technical support issues arising from supporting information (other than missing files) should be addressed to the authors.

Supplementary
