# Supplementary material for: Stabilization of an Enantiopure Sub‐monolayer of Helicene Radical Cations on a Au(111) Surface through Noncovalent Interactions
Source: Angew Chem Int Ed Engl. 2021 Jun 8;60(28):15276–80. doi: 10.1002/anie.202103710 (PMC8362206; doi:10.1002/anie.202103710)
Supplement: Supplementary file 1 — Supplementary [file ANIE-60-15276-s001.pdf]

Angewandte Chemie
Eine Zeitschrift der Gesellschaft Deutscher Chemiker

Supporting Information

Stabilization of an Enantiopure Sub-monolayer of Helicene Radical Cations on a Au(111) Surface through Noncovalent Interactions

*Niccolò Giaconi, Andrea Luigi Sorrentino, Lorenzo Poggini, Michela Lupi, Vincent Polewczyk, Giovanni Vinai, Piero Torelli, Agnese Magnani, Roberta Sessoli, Stefano Menichetti, Lorenzo Sorace, Caterina Viglianisi, and Matteo Mannini**

anie_202103710_sm_miscellaneous_information.pdf

Table of Contents

Figure S1. XPS N1*s* region of bulk **RadE** and **RadE_TP@Au**
Table S1. BE value of component of S2*p* region
Table S2. Most relevant peaks of positive ion ToF-SIMS spectra
Figure S2. Positive ion and negative ion ToF-SIMS spectra
Figure S3. EPR spectra of **RadE** solution at 200 K and at 110 K
Figure S4. Angular dependence of *g* value in function of the magnetic field orientation
Figure S5. XAS spectra of gold reference substrate

Experimental section

The substrate was obtained by evaporating gold on mica inside a vacuum chamber (≈ 10^{-6} mbar) with a deposition rate of 0.1 Å/s. A bulk reference sample was prepared by *drop-casting* using a 2 mM solution of **RadE** in dichloromethane. The molecular monolayer was assembled by incubating the H_2 flame annealed gold on mica substrate in a 2 mM solution of thiophenol in dry ethanol at room temperature. After 24h the surface was rinsed with pure ethanol and dried under nitrogen atmosphere. Besides, the thiols monolayer was immersed again in a 2 mM solution of **RadE** in dry CH_2Cl_2 for an additional 24h. Finally, the sample was rinsed with pure CH_2Cl_2 and dried under nitrogen atmosphere.

XPS measurements were performed using a micro-focused monochromatic Al K_α radiation source (1486.6 eV, model SPECS XR-MS Focus 600) and a multichannel detector electron analyzer (model SPECS Phoibos 150 1DLD) with a pass energy of 40 eV to ensure appropriate resolution. The spectra were measured in normal emission with the X-ray source mounted at 54.44° with respect to the analyzer. The binding energy scale was calibrated using the Au4$f_{7/2}$ peak at 84 eV. Spectra were deconvoluted using CasaXPS software introducing mixed Gaussian and Lorentzian contributions for each component. The background was fitted using the Shirley or linear method.

Tof-SIMS characterization was performed with a TRIFT III spectrometer (Physical Electronics, Chanhassen, MN) equipped with a gold liquid-metal primary ion source. Positive and negative ion spectra were acquired by rastering a pulsed, bunched 22 keV Au^+ primary ion beam over a 100 *µ*m X 100 *µ*m sample area maintaining static SIMS conditions. Positive-ion spectra were calibrated to $C_2H_5^+$ (*m/z* = 29.039), $C_7H_8^+$ (*m/z* = 92.063) and $C_7H_5S^+$ (*m/z* = 121.011). Negative-ion spectra were calibrated to CH^- (*m/z* = 13.008), F^- (*m/z* = 18.998) and $C_6H_6^-$ (*m/z* = 78.047). Mass resolution (*m/Δm*) was up to $5 \cdot 10^3$ depending on the sample.

CW X-band EPR spectra of the sample were recorded at 30 K on a Bruker Elexsys E500 spectrometer equipped with an SHQ cavity. EPR spectral simulation were performed using EasySpin.[1]

XNCD experiments were performed in total electron yield (TEY)[2] detection mode at APE-HE beamline[3] at Elettra synchrotron. This allowed us to both reach the extreme surface and element sensitivity required to investigate the low number of molecules constituting the monolayer and to work in reduced photon flux conditions to avoid radiation damage of the sample. Samples were prepared inside a glovebox under N_2 atmosphere and transferred to the fast entry of the chamber via a UHV suitcase to avoid any contact between the samples and the air, minimizing the atmospheric contamination. To remove the contribution of the adventitious carbon present in the beamline optical path each signal was normalized by using a reference XAS at Carbon K edge acquired on a freshly sputtered gold reference sample (see Figure S5): each measurement has been repeated twice, first monitoring the I/I_0 on the real sample and then repeating the same measurement of the gold single crystal, then a ratio between the two spectra has been adopted as the final spectrum for each polarization. In that way, the obtained absorption signal is attributable only to the carbon atoms of the sample. XNCD spectra has been evaluated as the difference between left and right circularly polarized light (σ^L-σ^R), by normalizing to 1 the average of the two XAS spectra at 290 eV (in the post edge energy region), and taking into account the degree of circular polarization of about 95%.

Results and Discussion

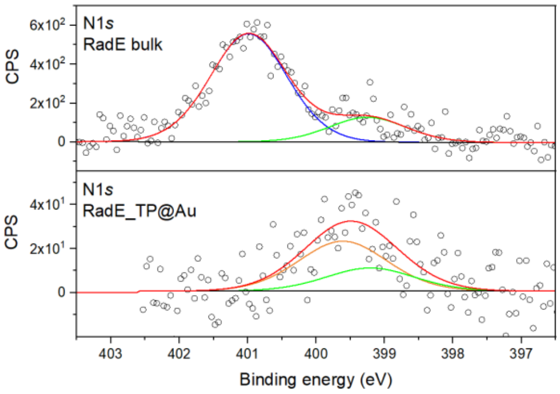

Figure S1. N1*s* region of bulk **RadE** and **RadE_TP@Au**. After the exposure to X-rays we can observe a shift of the main nitrogen component toward lower binding energy.

Table S1. Binding energy value (eV) of the XPS S2*p* region of bulk **RadE**, **TP@Au** and **RadE_TP@Au** samples.

S2$p_{3/2}$ region	Bulk	TP@Au	RadE_TP@Au
**S-Au**	----	161.3 (52.3 %)	161.1 (48.2 %)
**Thiophenol**	----	162.2 (32.2 %)	162.1 (22.5 %)
**RadE**	163.5 (100%)	----	163.6 (9.6 %)
**S$_{ox}$**	----	167.7 (15.5 %)	167.2 (19.7 %)

Table S2. Most relevant peaks in positive ion ToF-SIMS spectra recorded on bulk **RadE**, **TP@Au** and **RadE_TP@Au**

Assignment	Bulk	Int.	TP@Au	Int.	RadE_TP@Au	Int.
$[C_6H_6S]^+$			110	*w*	110	*m*
$[C_7H_5S]^+$	121	*vw*				
$[C_{14}H_{11}NS]^+$	225	*vw*			225	*m*
$[C_{14}H_{10}NS_2]^+$	256	*vw*			256	*w*
$[C_6H_5SAu]^+$			306	*s*	306	*w*
$[C_{21}H_{14}NS]^+$					312	*w*
$[C_{21}H_{16}NS]^+$	314	*w*				
$[C_{20}H_{12}NS_2]^+$	330	*m*			330	*w*
$[C_{21}H_8NS_2]^+$					338	*w*
$[C_{21}H_{17}NS_2]^+$	347	*s*			347	*w*
$[C_{21}H_{17}NOS_2]^+$	363	*w*				
$[C_{21}H_{15}NS_2F]^+$					364	*w*
$[C_{21}H_{15}NO_2S_2]^+$	377	*vw*				
$[C_{21}H_{15}NS_2F_2]^+$					383	*w*
$[C_{22}H_{15}NS_2F_2]^+$					395	*w*
$[C_{19}H_{13}AuNS_2]^+$	516	*vw*				
$[C_{21}H_{17}NS_2Au]^+$	544	*vw*				

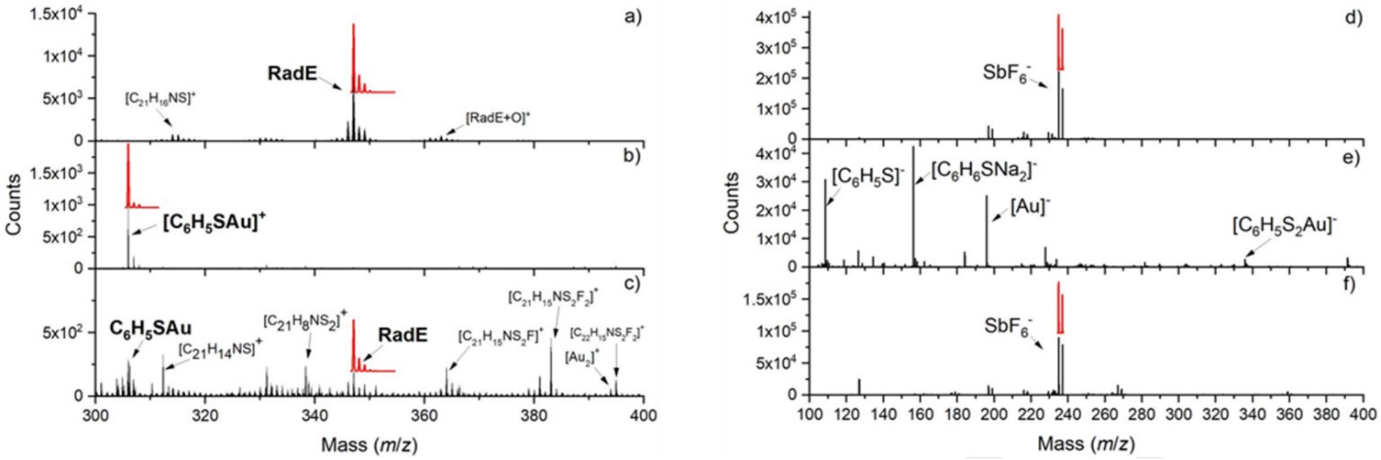

Figure S2. ToF-SIMS positive ion spectra of a) bulk **RadE**, b) **TP@Au** SAM and c) **RadE_TP@Au** in the region from 300 to 400 m/z. ToF-SIMS negative ion spectra of d) bulk **RadE**, e) **TP@Au** SAM and f) **RadE_TP@Au** in the region from 100 to 400 m/z. Red lines above the experimental data represent the theoretical isotopic distribution of the most significant peaks.

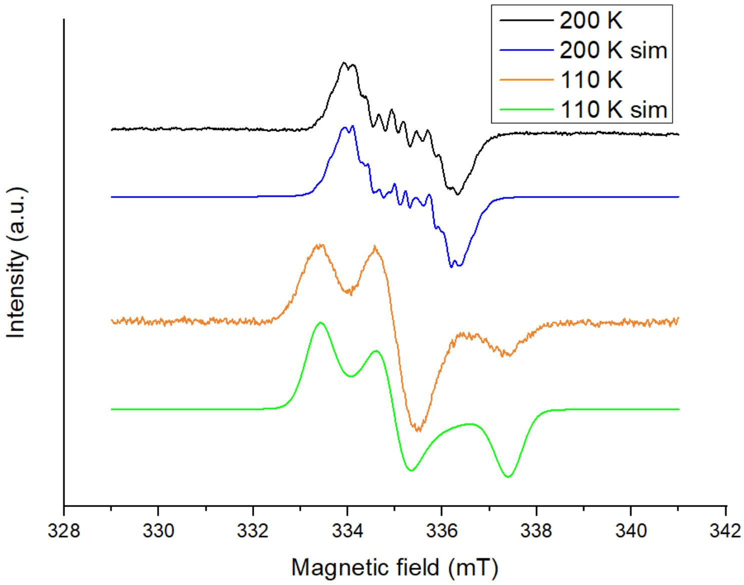

Figure S3. Experimental and simulated EPR spectra of **RadE** in solution (0.1 mM, dichloromethane/ethanol 3:1**)** above (black) and below (orange) freezing temperature.

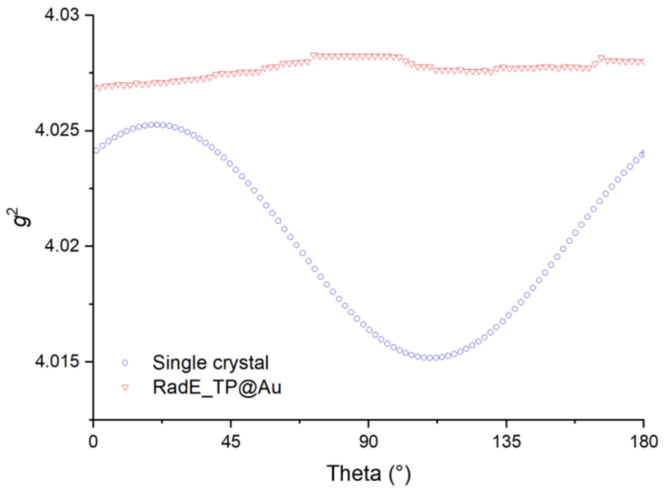

Figure S4. g^2 angular dependence of **RadE** single crystal around monoclinic b axis and **RadE_TP@Au**.

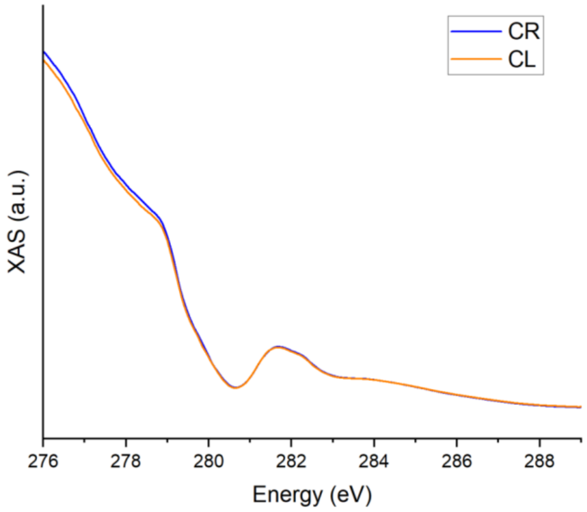

Figure S5. XAS spectra acquired at Carbon K edge on gold reference substrate.

References

[1] S. Stoll, A. Schweiger, *J. Magn. Reson.* **2006**, *178*, 42–55.
[2] B. L. Henke, J. Liesegang, S. D. Smith, *Phys. Rev. B* **1979**, *19*, 3004–3021.
[3] G. Panaccione, I. Vobornik, J. Fujii, D. Krizmancic, E. Annese, L. Giovanelli, F. Maccherozzi, F. Salvador, A. De Luisa, D. Benedetti, A. Gruden, P. Bertoch, F. Polack, D. Cocco, G. Sostero, B. Diviacco, M. Hochstrasser, U. Maier, D. Pescia, C. H. Back, T. Greber, J. Osterwalder, M. Galaktionov, M. Sancrotti, G. Rossi, *Rev. Sci. Instrum.* **2009**, *80*, 043105.

Author Contributions

NG, ML, CV and SM prepared the RadE system and the enantiopure analogues, NG, LP and MM designed the film architecture, prepared the monolayer deposits and performed the XPS experiments, NG and LS collected the EPR data, NG, LP, ALS, AM and MM participated to the ToF-SIMS experiments, NG, ALS, GV, VP, PT, LP, RS and MM designed, performed and discussed the XNCD experiments, NG, LP, LS and MM drafted the manuscript, all authors have contributed and approved the final version of the manuscript.
